# Understanding the Perioperative Perception of Pain in Patients with Crohn’s Disease: Epidural Versus Non-Epidural Analgesia

**DOI:** 10.3390/jcm14124383

**Published:** 2025-06-19

**Authors:** Regina Pistorius, Anna Widder, Marleen Sabisch, Christian Markus, Michael Meir, Imad Maatouk, Christoph-Thomas Germer, Patrick Meybohm, Nicolas Schlegel, Matthias Kelm, Sven Flemming

**Affiliations:** 1 Department of General, Visceral, Transplant, Vascular and Pediatric Surgery, University Hospital Würzburg, 97080 Würzburg, Germany; 2 Department of Anaesthesiology, Intensive Care, Emergency and Pain Medicine, University Hospital Würzburg, 97080 Würzburg, Germany; 3 Department of Internal Medicine, Division of Psychosomatic, Psychotherapy and Psychooncology, University Hospital Würzburg, 97080 Würzburg, Germany

**Keywords:** colorectal surgery, Crohn’s disease, pain management, perioperative medicine

## Abstract

**Background:** Patients with Crohn’s disease (CD) suffer from a relevant burden of abdominal pain and psychological distress that can aggravate postoperatively. While systematic strategies for postoperative pain management are lacking, the potential benefit of perioperative epidural analgesia (EDA) in CD patients is unclear. **Methods**: All patients receiving an ileocecal resection due to CD at a tertiary hospital were included. The impact of epidural versus non-epidural analgesia on postoperative pain perception was evaluated by analyzing the numeric rating scale (NRS), analgesic consumption, and clinical outcomes. **Results:** In this monocentric study, 172 patients receiving ileocecal resection due to CD were included, with 122 receiving EDA. The epidural pain catheters were kept for an average of 4.4 days (±1.3) before being removed. EDA resulted in significantly decreased pain as well as a decreased amount of analgesic consumption (adjuvant analgesics: 16.4% vs. 32%, *p* = 0.021; strong opioids: 30.3% vs. 72.0%, *p* < 0.001) at the early postoperative course (1 vs. 3 at rest and 2 vs. 4 movement-evoked, *p* < 0.001). No difference in pain perception was detected on day 5 between EDA and non-EDA patients. Patients with EDA had a significantly longer length of hospital stay (7.5 versus 6 days, *p* = 0.002) and an increased intake of weak opioids at discharge (*p* = 0.024). **Conclusions:** While EDA in CD patients resulted in significantly decreased pain and decreased amounts of analgesic adjuvants and strong opioids at the early postoperative course, intravenous and oral analgesia provide sufficient postoperative pain control after surgery and earlier patient autonomy.

## 1. Introduction

Crohn’s disease (CD) represents a significant socioeconomic burden and poses substantial challenges to healthcare systems worldwide [1,2]. As a chronic, lifelong condition characterized by alternating periods of relapse and remission, CD can severely impact patients’ physical, mental, and social well-being, leading to a reduced overall quality of life [3]. Current evidence and guidelines suggest one to consider ileocecal resection (ICR) early in cases of localized terminal ileitis, underscoring the persistent need for surgical intervention despite medical progress [4,5].

Acute postoperative pain is a frequent issue following surgery, with a significant proportion of patients experiencing severe pain [6,7]. Postoperative pain is debilitating and one of the most common complications after surgery, often leading to personal suffering, delayed functional recovery, prolonged hospital stays [8], and an increased risk for chronic postsurgical pain [9,10]. McKevitt et al. demonstrated that CD patients experience significantly more pain and require more postoperative analgesia, as well as displaying an enhanced inflammatory response following elective laparoscopic right hemicolectomy, compared to patients with neoplasia undergoing the same operation performed by the same surgeon [11]. As a result, postoperative pain management in these patients is a critical aspect of recovery, influencing both short-term outcomes, such as mobilization and the length of hospital stay, and long-term quality of life. Effective pain management in CD patients is complex due to the nature of the disease and the need for tailored approaches that consider both the inflammatory process and surgical trauma.

Perioperative thoracic epidural analgesia (EDA) is recommended as first-line treatment after major (open) abdominal surgeries by current guidelines [12,13,14]. Compared to general anesthesia alone, the combination of epidural and general anesthesia has been shown to decrease the requirement of general anesthetics [15], enhance postoperative pain relief, and reduce opioid use [13,16]. Additionally, EDA improves postoperative pain management, minimizes pulmonary complications, shortens the duration of postoperative ileus, and hospital length of stay after major abdominal surgery compared to opioid therapy alone [17,18]. However, the controversial evidence for the effects of EDA on morbidity and mortality in abdominal surgery remains a subject of ongoing debate [19,20,21,22].

The potential benefit of perioperative EDA in CD patients undergoing ileocecal resection is unclear. To improve postoperative care and patient outcomes in CD surgery, we investigate the impact of epidural versus non-epidural analgesia on postoperative pain perception by analyzing the numeric rating scale (NRS), pain scores, analgesic consumption, and clinical outcomes such as the length of hospital stay and postoperative complications for all consecutive patients who received ileocecal resection due to CD at our hospital.

## 2. Materials and Methods

### 2.1. Study Population

All patients who underwent ileocecal resection due to CD at the Department of General, Visceral, Transplant, Vascular, and Pediatric surgery at the University Hospital Würzburg, Germany, between 2017 and 2023, were included in this study. A multidisciplinary team, including a surgeon, a gastroenterologist, and a radiologist, determined the indication for an operation. All patients were divided into two groups based on the perioperative pain management: general anesthesia with postoperative intravenous analgesia and oral analgesics or general anesthesia with postoperative EDA. Operations were performed by three experienced colorectal surgeons specialized in IBD surgery. Clinical data, including patient baseline characteristics (age, sex, ASA score), co-morbidities, and their duration of hospital stay, were collected for each patient from patient records. The ASA classification defines the physical status of the patient and is used to predict perioperative risks (I = healthy, II = mild systemic disease, III = severe systemic disease, IV = disease that is a threat to life). Additionally, surgical data, including the type of surgery, operating time, and complications according to the Clavien–Dindo classification (CDC), were retrieved from the local prospectively recorded database. Furthermore, postoperative need for pain medication and pain severity both at rest and with movement were assessed using the numeric rating scale (NRS; Likert scale 1–10, where 1 denotes no pain and 10 the worst pain).

### 2.2. Outcome

The primary outcome measure was postoperative pain scores on days 1, 3 and 5, assessed by using the NRS that was noted on the patient record, ranging from 1 (no pain) to 10 (worst imaginable pain ever). Secondary endpoints were the length of hospital stay as well as the postoperative analgesic score (postoperative days 3 and 5 and discharge). As Widder et al. demonstrated, the analgesic score is a simple and reliable measurement tool to relate pain and the necessary pain medication while avoiding misjudgments [23,24]. According to their potency based on the WHO analgesics ladder, the analgesics were defined as non-opioids, weak opioids, and strong opioids. Each substance or dose taken was assigned a scoring using an ascending point scale. To quantify pain with this scale, an analgesic score could be calculated. In addition, postoperative complications within 30 days were analyzed (Clavien–Dindo classification), as well as the use of adjuvant analgesics such as antidepressants and benzodiazepines.

### 2.3. Statistical Analysis

Statistical analysis was performed using IBM SPSS Statistics for Windows, version 28.0 (IBM Corp., Armonk, NY, USA). The distribution of variables was examined for normality using the Kolmogorov–Smirnov test. Descriptive data are presented as mean with standard deviation (SD) or median with range or total numbers with percentage. Differences in patient characteristics were assessed by the Chi-squared test and *t*-test or Mann–Whitney U-test, according to data scale and distribution. Statistical relevance was considered for a *p*-value < 0.05.

### 2.4. Ethical Approval

Ethical approval for this study was obtained from the Ethics Committee of the University of Würzburg, Germany (Approval number: 20230531 02).

## 3. Results

### 3.1. Patient Cohort

In this single-center study, 172 patients received ileocecal resection due to CD at the Department of Surgery at the University Hospital of Wuerzburg. Of those, 50 patients were assigned to general anesthesia with postoperative intravenous analgesia alone. In contrast, 122 patients received general anesthesia in combination with EDA, particularly if an open approach was planned (18% versus 41%). As presented in Table 1, both groups did not differ in terms of patient characteristics such as age, BMI, smoking habits, ASA classification, Charlson Comorbidity Index (CCI), or their preoperative levels of hemoglobin and albumin. Further analysis revealed no significant differences for preoperative self-administered pain medication, including adjuvant analgesics between both patient groups (Table S1). However, a significant difference was observed between both patient groups regarding the surgical approach. While 72 (59.0%) patients with a combined general anesthesia and perioperative thoracic EDA received a laparoscopic approach, 40 (80.0%) patients with a general anesthesia alone received laparoscopic ICR (*p* = 0.006). In 50 (41.0%) patients with EDA and 9 (18.0%) patients without EDA, an open approach for ICR was performed. The epidural pain catheters were kept for an average of 4.4 days (±1.3) before being removed.

### 3.2. Postoperative Outcome

**Figure 1:**
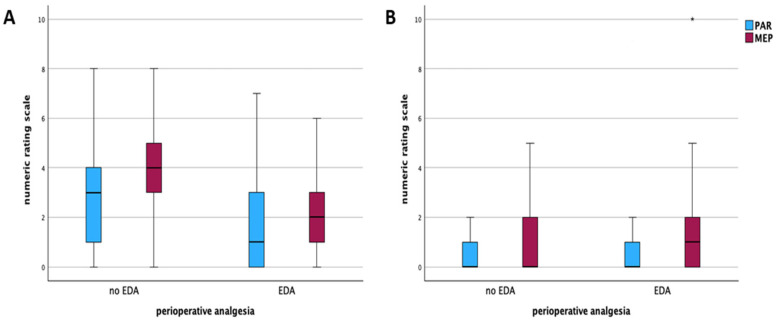
Perception of pain on postoperative day 1 (PAR: *p* < 0.001, MEP: *p* < 0.001) (**A**) and day 5 (PAR: *p* = 0.533, MEP: *p* = 0.270) (**B**), measured with NRS compared between patients with intravenous analgesia alone and patients with combined analgesia (epidural pain catheter and intravenous analgesia). PAR = pain at rest. MEP = movement-evoked pain. EDA = epidural analgesia. NRS = numeric rating scale. * = statistical significantly different.

Regarding the primary outcome, patients with general anesthesia and postoperative intravenous analgesia and oral analgesics alone had significantly higher pain scores (NRS) on postoperative day 1 (1 vs. 3 at rest and 2 vs. 4 movement-evoked, *p* < 0.001). However, no differences regarding pain at rest (PAR) or movement-evoked pain (MEP) were observed between both groups on any following postoperative day (Table 2, Figure 1). The postoperative complications detected and evaluated using the Clavien–Dindo classification demonstrated a low CCI for both groups (4.4 versus 0.0, *p* = 0.79), as well as similar rates of severe complications (Clavien–Dindo > IIIa) (13 versus 6, *p* = 0.80). In addition, no differences were seen for rates of anastomotic leakage (7 versus 5, *p* = 0.32), postoperative ileus (21 versus 5, *p* = 0.23), or rates of wound infections (18 versus 9, *p* = 0.60) (Table 3). Length of hospital stay was significantly shorter for patients receiving only systemic pain medication compared to patients who perioperatively received an epidural pain catheter (7.5 versus 6 days, *p* = 0.002) (Table 1).

Complications from the epidural pain catheter were few and did not pose any permanent deficits. Of the 122 catheters placed, only 1 (0.8%) was associated with a major complication (epidural empyema which needed surgical intervention). We found minor complications such as unobserved disconnection, paresthesia, or the dislodging of the catheter in 8 (6.7%) patients. Two (1.2%) patients showed eczema around the injection site. All these findings resulted in the removal of the epidural catheter by the acute pain service.

### 3.3. Analgesic Consumption

**Figure 2:**
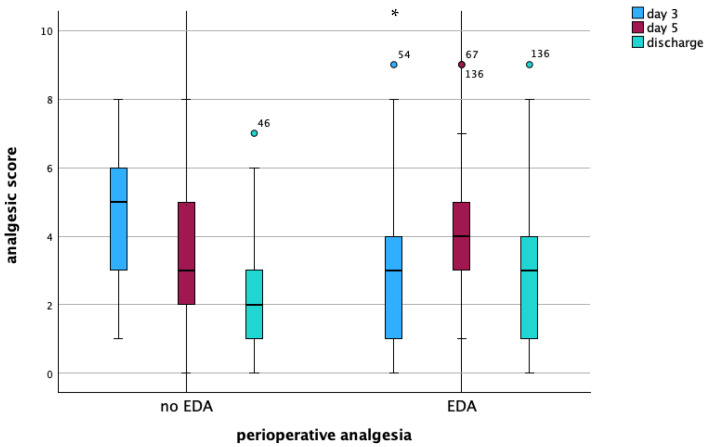
Analgesic score compared between patients with intravenous analgesia alone and patients with combined analgesia (epidural pain catheter and intravenous analgesia) on postoperative day 3 (*p* * < 0.001), on day 5 (*p* = ns), and at discharge (*p* = ns). EDA = epidural analgesia.

Overall, 36 (20.9%) CD patients who underwent ICR received adjuvant analgesics. Patients with combined EDA received significantly lower amounts of adjuvant analgesics (20 (16.4%) vs. 16 (32%), *p* = 0.021, Table 4). The consumption of strong opioids during the postoperative stay was significantly lower in patients with EDA compared to intravenous analgesia and oral analgesics alone (30.3% vs. 72.0%, *p* < 0.001). However, while the analgesics score was significantly higher for non-EDA patients compared to EDA patients on postoperative day 3 (5 versus 3, *p* = 0.001), scores equalized on postoperative day 5 (4 versus 4, *p* = 0.536) (Table 5, Figure 2). At discharge, 57 (46.7%) patients with EDA and 14 (28.0%) patients without EDA received weak opioids at discharge (*p* = 0.024) (Table 6).

In a subgroup analysis, patients with minimal invasive surgery only were included and analyzed regarding the postoperative pain perception (Table S2). Similar to the overall cohort, patients with minimal invasive surgery and EDA had significantly reduced pain scores on day 1 compared to non-EDA patients, but this effect was eliminated at a later postoperative stage on day 3 and 5, when pain scores were comparable between both cohorts at rest and movement (Table 7). In addition, the analgesics score demonstrated that patients without EDA had significantly higher scores at the early postoperative course (day 3); however, no significant differences could be detected at the day of discharge (3 versus 1, *p* = 0.057, Table S3).

## 4. Discussion

Mental health and pain perception are important aspects, not only postoperatively but in general. However, while CD patients often suffer from increased chronic abdominal pain and psychological burden compared to non-CD patients [25], the symptoms can significantly aggravate following surgery. Since surgery is an important factor in the interdisciplinary treatment management for CD and rates of surgery remain relevant over time, sufficient perioperative strategies are necessary to reduce psychological distress and pain while improving patient outcome. Here, we report for the first time a structured analysis of postoperative approaches on pain management in CD patients. While epidural analgesic catheters (EDA) decrease pain perception during early postoperative days, this effect is reversed after five days and was associated with an increased length of hospital stay and opioid consumption at discharge.

In this monocentric study, 172 patients receiving ileocecal resection due to CD were included, resulting in a homogenous patient cohort with patient characteristics being widely comparable between the two groups. To investigate postoperative pain sensation, the established NRS and analgesics score were used, which have been shown to reliably correlate with the individual perception of pain and the need for analgesia [23]. When analyzing the results from different aspects, EDA led to significantly decreased pain as well as a decreased amount of analgesic consumption at the early postoperative course, despite the fact that significantly more patients of the EDA cohort were operated on by an open approach. NRS scores for rest and movement were overall low for both groups on postoperative day 1, thus demonstrating the effective pain management independently of the application (NRS 1–4). However, no effect of EDA could be detected on day 5, when the amount of epidural analgesia is usually reduced and catheters are removed, resulting in comparable levels of pain reported. Importantly, the subgroup analysis of patients being operated on minimally invasively confirmed that the observed effect is independent of the surgical approach (Table 7). Moreover, the analysis revealed that patients with EDA had a significantly enhanced length of hospital stay (7.5 versus 6 days, *p* = 0.002) and an increased intake of weak opioids at discharge 46.7% versus 28.0%, *p* = 0.024). While on the one hand, an effect of the surgical approach (open surgery: 18% versus 41%) cannot be excluded as a partial explanation for an enhanced hospital stay, an epidural catheter in place might reduce the mobilization of patients, and difficulties in switching EDA to oral analgesics may need more time to adjust, thus resulting in a delayed readiness for discharge. Therefore, this study demonstrates that the initially positive effect of EDA at the early postoperative course might be hindered by a longer hospital stay (median 1.5 days), thus suggesting equal sufficiency and potential economic advantages of non-EDA pain management in the cohort of CD patients.

IBD patients suffer from a chronic intestinal disorder often accompanied by a relevant burden of abdominal pain and psychological distress [25,26]. Despite that, systematic analysis and recommendations on the specific perioperative pain management in IBD patients are lacking, although enhanced recovery programs are well-established [27]. In a small retrospective single-center study including only 38 patients, McKevitt et al. reported an increased postoperative pain perception of CD patients compared to non-CD patients [11]. Similarly, another small study with 20 patients demonstrated that intraoperative opioid requirements are significantly higher for patients suffering from IBD [28]. Those observations were confirmed by Huehne et al., but the authors failed to detect differences in pain sensitivity for CD patients, thus speculating on a more complex pathway as potential modulator [29]. In line with that, various studies have demonstrated the potential of psychological interventions on pain reduction in CD patients, underlining the complex etiology of pain perception in CD patients [30,31]. Indeed, chronic psychological distress and differences in general pain perception might be crucial aspects for the results of previous studies on perioperative pain experiences in patients with and without CD. In line with that, a relevant number of CD patients of our cohort received adjuvant analgesics during the postoperative course (20.9%). However, despite the great socioeconomic relevance in the large cohort of IBD patients, specific investigations on pain management strategies in this complex patient cohort beyond the few small studies reported are lacking. To date, no established pain management strategies exist for IBD patients, with many of the concepts applied being ineffective or even detrimental [26]. To address this aspect, we analyzed for the first time a large homogenous cohort of CD patients on the postoperative effectiveness and outcome of different pain management strategies. Based on our robust evidence, we demonstrate that EDA provides positive effects in the early postoperative course until days 3–5. However, sufficient intravenous and oral analgesia results in adequate pain relief for CD patients postoperatively, a shorter length of hospital stay and decreased need for pain medication at discharge. Importantly, a subgroup analysis of patients receiving minimal invasive surgery only confirmed those results, thus underlining that pain perception in CD patients is largely independent of the surgical approach (Table 7).

Our work has some limitations, including the lack of randomization and blinding. Furthermore, the study has a retrospective design, and all patients were only referred from a single tertiary center. However, our analysis is the first study investigating postoperative pain management in CD patients, and the cohort represents a homogeneous group of patients, representing daily clinical routine. Therefore, our results provide significant evidence and direct impact on the postoperative management of IBD patients who relevantly suffer from abdominal pain and psychological distress.

## 5. Conclusions

Abdominal pain and psychological burden relevantly affect patients suffering from CD, and those complaints often aggravate postoperatively. We provide novel evidence to better understand pain perception in CD patients. While EDA resulted in significantly decreased pain and the need for pain medication at the early postoperative course, intravenous and oral analgesia provide sufficient postoperative pain control after surgery combined with earlier patient autonomy in the specific cohort of patients suffering from CD. Future studies should also focus on the additional effect of psychological interventions due to the complex etiology of pain perception in CD patients.

## Figures and Tables

**Table 1:** Patient characteristics.

	All (n = 172, 100%)	EDA (n = 122, 70.9%)	No EDA (n = 50, 29.1%)	*p*-Value
gender, n (%)				
male	90 (52.3%)	65 (53.3%)	25 (50%)	0.696
female	82 (47.7%)	57 (46.7%)	25 (50%)	
age (years), mean ± SD	38.2 ± 14.9	37.5 ± 15.1	39.8 ± 14.5	0.350
BMI (kg/m^2^), mean ± SD	24.2 ± 4.7	23.7 ± 4.4	25.3 ± 5.4	0.052
active smoking, n (%)	49 (28.5%)	30 (24.6%)	19 (38.0%)	0.077
ASA classification, n (%)				0.338
I	12 (7%)	10 (8.2%)	2 (4.0%)	
II	154 (89.5%)	109 (89.3%)	45 (90.0%)	
III	6 (3.5%)	3 (2.5%)	3 (6.0%)	
Charlson Comorbidity Index (CCI), median	0 [0; 1]	0 [0; 1]	0 [0; 1]	0.325
albumin (g/dl), mean ± SD	4.2 ± 0.5	4.2 ± 0.4	4.1 ± 0.6	0.556
haemoglobin (g/dl), mean ± SD	13.0 ± 1.9	13.0 ± 1.7	13.0 ± 2.2	0.968
depression, n (%)	11 (6.4%)	6 (4.9%)	5 (10.0%)	0.216
surgical approach, n (%)				**0.006**
laparoscopic	122 (65.1%)	72 (59.0%)	40 (80.0%)	
robotic-assisted	1 (0.6%)	0 (0%)	1 (2.0%)	
open	59 (34.3%)	50 (41.0%)	9 (18.0%)	
stoma	13 (7.6%)	12 (9.8%)	1 (2.0%)	0.077
length of hospital stay (days), median [range]	7 (6; 9) (3; 43)	7.5 (6; 9) (5; 41)	6 (5; 9) (3; 43)	**0.002**

EDA, epidural analgesia. BMI, body mass index.

**Table 2:** Postoperative outcome.

	All (n = 172, 100%)	EDA (n = 122, 70.9%)	No EDA (n = 50, 29.1%)	*p*-Value
NRS day 1 PAR, median	2 [0; 3]	1 [0; 3]	3 [1; 5]	**<0.001**
NRS day 1 MEP, median	3 [1; 4]	2 [1; 3]	4 [3; 6]	**<0.001**
NRS day 3 PAR, median	1 [0; 2]	1 [0; 2]	1 [0; 2]	0.685
NRS day 3 MEP, median	2 [0; 3]	1 [0; 3]	2 [0; 3]	0.659
NRS day 5 PAR, median	0 [0; 1]	0 [0; 1]	0 [0; 1]	0.533
NRS day 5 MEP, median	1 [0; 2]	1 [0; 2]	0 [0; 2]	0.270

EDA, epidural analgesia. NRS, numeric-rating scale. PAR, pain at rest. MEP, movement-evoked pain.

**Table 3:** Postoperative complications until postoperative day 30.

	All (n = 172, 100%)	EDA (n = 122, 70.9%)	No EDA (n = 50, 29.1%)	*p*-Value
complications, n (%)				
ileus	26 (15.1%)	21 (17.2%)	5 (10.0%)	0.230
anastomotic leakage	12 (7.0%)	7 (5.7%)	5 (10.0%)	0.319
re-operation	20 (11.6%)	13 (10.7%)	7 (14.0%)	0.534
wound infection	27 (15.7%)	18 (14.8%)	9 (18.0%)	0.595
Clavien–Dindo > 3a	19 (11.0%)	13 (10.7%)	6 (12.0%)	0.798
Clavien–Dindo CCI, median (range)	0 (0; 21) (0; 85)	4.4 (0; 21) (0; 71)	0 (0; 21) (0; 85)	0.793

EDA, epidural analgesia.

**Table 4:** Postoperative analgesic consumption until discharge.

	Overall (N = 172)	EDA (N = 122)	No EDA (N = 50)	*p*-Value
psychiatric counseling, n (%)	3 (1.7%)	1 (0.8%)	2 (4.0%)	0.150
adjuvant analgesic, n (%)	36 (20.9%)	20 (16.4%)	16 (32.0%)	**0.021**
adjuvant analgesic duration, days	4.5 (1; 8)			
Novalgin, n (%)	151 (87.8%)	108 (88.5%)	43 (86.0%)	0.668
Paracetamol, n (%)	118 (68.6%)	78 (63.9%)	40 (80.0%)	0.094
strong opioid, n (%)	73 (42.4%)	37 (30.3%)	36 (72.0%)	**<0.001**

EDA, epidural analgesia.

**Table 5:** Analgesic score.

	All (n = 172, 100%)	EDA (n = 122, 70.9%)	No EDA (n = 50, 29.1%)	*p*-Value
analgesic score day 3, median [range]	3 [1; 5] [0; 9]	3 [1; 4] [0; 9]	5 [3; 6] [0; 9]	**<0.001**
analgesic score day 5, median [range]	4 [3; 5] [0; 9]	4 [3; 5] [1; 9]	3 [2; 5] [0; 8]	0.536
analgesic score at discharge, median [range]	3 [1; 3] [0; 9]	3 [1; 4] [0; 9]	2 [1; 3] [0; 7]	0.054

EDA, epidural analgesia.

**Table 6:** Pain medication at discharge.

	All (n = 172, 100%)	EDA (n = 122, 70.9%)	No EDA (n = 50, 29.1%)	*p*-Value
adjuvant analgesic, n (%)	8 (4.7%)	4 (3.3%)	4 (8.0%)	0.182
Novalgin, n (%)	108 (62.8%)	74 (60.7%)	34 (68.0%)	0.366
Paracetamol, n (%)	87 (50.6%)	60 (49.2%)	27 (54.0%)	0.566
Ibuprofen, n (%)	4 (2.3%)	2 (1.6%)	2 (4.0%)	0.351
weak opioid, n (%)	71 (41.3%)	57 (46.7%)	14 (28.0%)	**0.024**
strong opioid, n (%)	23 (13.4%)	18 (14.8%)	5 (10.0%)	0.405

EDA, epidural analgesia.

**Table 7:** Postoperative outcome of patients with minimal invasive surgery only.

	All (n = 113, 100%)	EDA (n = 72, 63.7%)	No EDA (n = 41, 36.3%)	*p*-Value
NRS day 1 PAR, median [quartile] [range]	2 [0; 3] [0; 8]	1 [0; 3] [0; 8]	3 [1; 4] [0; 8]	**0.009**
NRS day 1 MEP, median [quartile] [range]	3 [1; 4] [0; 8]	2 [1; 3] [0; 8]	4 [2.5; 5] [0; 8]	**<0.001**
NRS day 3 PAR, median [quartile] [range]	1 [0; 2] [0; 7]	1 [0; 2] [0; 7]	1 [0; 2] [0; 7]	0.902
NRS day 3 MEP, median [quartile] [range]	2 [0; 3] [0; 7]	1 [0; 3] [0; 7]	2 [0; 3.5] [0; 6]	0.756
NRS day 5 PAR, median [quartile] [range]	0 [0; 1] [0; 9]	0 [0; 1] [0; 9]	0 [0; 1] [0; 5]	0.360
NRS day 5 MEP, median [quartile] [range]	1 [0; 2] [0; 10]	1 [0; 2] [0; 10]	0 [0; 2] [0; 6]	0.372

Data are median [quartiles] [range], and *p*-values were computed using Mann–Whitney U-test as appropriate. EDA, epidural analgesia. NRS, numeric-rating scale. PAR, pain at rest. MEP, movement-evoked pain.

## Data Availability

The data presented in this study are available on request from the corresponding author.

## Supplementary Materials

The following supporting information can be downloaded at: https://www.mdpi.com/article/10.3390/jcm14124383/s1, Table S1: Preoperative self-administered pain medication, n [%]; Table S2: Patient characteristics of patients with minimal invasive surgery only, n [%]; Table S3. Analgesics score of patients with minimal invasive surgery only.
