# Supplementary material for: Understanding the Perioperative Perception of Pain in Patients with Crohn’s Disease: Epidural Versus Non-Epidural Analgesia
Source: J Clin Med. 2025 Jun 19;14(12):4383. doi: 10.3390/jcm14124383 (PMC12193785; doi:10.3390/jcm14124383)
Supplement: Supplementary file 1 [file jcm-14-04383-s001.zip › jcm-3677658-supplementary.pdf]

Supplementary Materials

Suppl. Table S1. *Preoperative self-administered pain medication, n [%].*

	all (n=172, 100%)	EDA (n=122, 70.9%)	no EDA (n=50, 29.1%)	P-Value
adjuvant analgesics	14 (8.1%)	11 (9.0%)	3 (6.0%)	0.511
self-administered pain medication	13 (7.6%)	8 (6.6%)	5 (10.0%)	0.438
non-opioids	10 (5.8%)	6 (4.9%)	4 (8.0%)	0.433
weak opioids	2 (1.2%)	1 (0.8%)	1 (2.0%)	0.512
strong opioids	2 (1.2%)	1 (0.8%)	1 (2.0%)	0.512

EDA, epidural analgesia.

Suppl. Table S2. *Patient characteristics of patients with minimal invasive surgery only, n [%].*

	all (n=113, 100%)	EDA (n=72, 63.7%)	no EDA (n=41, 36.3%)	P-Value
gender, n [%]				
male	48 (42.5%)			
female	65 (57.5%)			
age [years], mean	35.4 ± 13.6	33.5 ±13.1	38.7 ± 14.0	0.052
BMI [kg/m^2], mean	23.8 ± 4.4	23.2 ± 3.9	24.8 ± 5.1	0.087
active smoking, n [%]	30 (26.5%)	14 (19.4%)	16 (39%)	0.023
ASA classification, n [%]				0.333
I	8 (7.1%)	6 (8.3%)	2 (4.9%)	
II	104 (92.0%)	66 (91.7%)	38 (92.7%)	
III	1 (0.9%)	0	1 (2.9%)	
Charlson Comorbidity Index (CCI), median [quartile] [range]	0 [0;0] [0;3]			
albumin [g/dl], mean	4.2 ± 0.5	4.2 ± 0.4	4.1 ± 0.6	0.389
haemoglobin [g/dl], mean	12.9 ± 2.0	12.9 ± 1.9	12.8 ± 2.3	0.719
depression, n [%]	7 (6.2%)	3 (4.2%)	4 (9.8%)	0.236
stoma	3 (2.7%)	3 (4.2%)	0	0.185
length of hospital stay [days], median [quartile] [range]	8 [6 ;8] [3; 43]	7 [6; 8] [5; 41]	6 [4.5; 8.5] [3; 43]	**0.018**

Data are n (%), mean ± sd or median [quartiles] [range] as appropriate. p-values were computed using X^2 -test, student's t-test or Mann-Whitney U-test as appropriate. EDA = epidural analgesia. BMI, body mass index.

Suppl. Table S3. *Analgesics score of patients with minimal invasive surgery only.*

	all (n=113, 100%)	EDA (n=72, 63.7%)	no EDA (n=41, 36.3%)	P-Value
analgesic score day 3, median [quartile] [range]	3.5 [2; 5] [0; 9]	3 [1; 4] [0; 9]	5 [2.5; 6] [0; 9]	**< 0.001**
analgesic score day 5, median [quartile] [range]	3.6 [3; 5] [0; 9]	4 [3; 4] [1; 9]	3 [2; 5] [0; 8]	0.108
analgesic score at discharge, median [quartile] [range]	2.4 [1; 3] [0; 9]	3 [1; 3] [0; 9]	1 [1; 3] [0; 7]	0.057

Data are median [quartiles] [range] and p-values were computed using Mann-Whitney U-test as appropriate. EDA = epidural analgesia.
